# Barriers experienced by community midwives to provide basic emergency obstetric and newborn care in rural Pakistan

**DOI:** 10.1186/s12913-023-10273-5

**Published:** 2023-11-27

**Authors:** Ramesh Kumar, Vikram Mehraj, Jamil Ahmed, Shahzad Ali Khan, Tariq Mehmood Ali, Saima Batool, Fozia Rashid, Sathirakorn Pongpanich

**Affiliations:** 1 Health Services Academy, Islamabad, Pakistan; 2 College of Public Health Sciences, Chulalongkorn University, Bangkok, Thailand; 3 McGill University Health Centre, Montreal, QC H4A 3J1 Canada; 4 Department of Family and Community Medicine, College of Medicine and Medical Sciences, Arabian Gulf University, Manama, Bahrain; 5 College of Nursing, Pishin Baluchistan, Pakistan; 6 Shifa International Hospital, Islamabad, Pakistan

**Keywords:** Barriers, Work environment, Permanence, Maternal care and community midwives, Pakistan

## Abstract

**Background:**

Considering the high maternal mortality rate, the government of Pakistan has deployed Community Midwives (CMWs) in rural areas of Pakistan. This relatively new cadre of community-based skilled birth attendants has previously reported to experience several challenges in providing maternal and child healthcare. However, what barriers they experience in providing basic emergency obstetric and newborn care needs to be further studied.

**Methods:**

This was a cross-sectional study conducted in twelve districts in Sindh province, Pakistan, with poor maternal and child health indicators. A total of 258 CMWs participated in this study and completed the questionnaire on a pretested, validated tool in their community-based stations. The trained data collectors completed the questionnaires from the respondents. The problems identified were categorized into three major issues: financial, and transport and security related; and were analyzed accordingly. Ethical approval was obtained from the institutional review board (IRB) of Health Services Academy (HSA) Islamabad, Pakistan.

**Results:**

The majority (90%) of 258 CMWs had formal training in maternal and neonatal care from the recognized institutions. Financial difficulties faced by CMWs were identified as the most frequent barriers and others were transport, security, and other issues. In univariate analysis, 38.1% and 61.9% of the community midwives who faced financial difficulties had completed a graduation or intermediate level of education, respectively (p = 0.006). Round-the-clock availability for emergencies was inversely associated with having financial difficulties, i.e., 71.4%, in contrast to 28.4% who had financial difficulties were available round-the-clock for emergency calls in their community clinics (p = 0.008). Formal training (p = 0.001), work experience (p = 0.015), longer duration of work (p = 0.003), and liaison with health workers and posting district (p = 0.001) had statistically significantly higher transport related issues. Security difficulties faced by CMWs and a set of correlates such as formal training (p = 0.019), working experience (p = 0.001), longer duration of work (p = 0.023), 24 h of availability on call (p = 0.004), liaison with traditional birth attendants (TBAs) in the community (p = 0.002), and district of posting (p = 0.001) were statistically significantly different. Other issues like working experience (p = < 0.001) and Liaison with TBAs in the community (p = < 0.001) were found statistically significant.

**Conclusion:**

Financial, transportation and security related barriers were commonly reported by community midwives in the delivery of basic emergency obstetric and newborn care in rural Pakistan.

## Introduction

Globally, maternal mortality is considered a serious public health problem, with a maternal mortality ratio of 159 per 100,000 live births [1]. Each year, around 303, 000 mothers die due to complications during pregnancy and birth [2]. In comparison to the developed world, low-income countries contribute 99% to maternal deaths [3]. Multiple factors like fragile health systems, poor access, and availability of emergency obstetric and newborn care (EmONC) contribute to a higher number of maternal deaths in developing countries [4]. EmONC is specialized care to reduce maternal and neonatal mortality as recommended by the World Health Organization (WHO) [3]. Trained healthcare workers could avert a substantial number of deaths through the provision of skilled care during the antenatal period and around birth [1–3].

Maternal and child health burden continues to be high in Pakistan despite the increased allocation of funds by public and donor agencies during the last decade [5]. Although the country has made significant improvement in reducing maternal mortality, it failed to achieve the millennium development goals 4 and 5 by 2015 [6]. This failure was partially attributed to a low number of skilled birth attendants, especially in rural communities. Skilled birth attendants provide basic care during pregnancy and birth [7]. However, births continue to be attended by unskilled persons in Pakistan, and home births are still favored. According to a recent survey, 40% of births in rural areas are attended by traditional birth attendants [8]. About 58% of births are reported to happen in homes in rural areas, with 10% and 31% occurring at public health and private health facilities, respectively [9].

The government of Pakistan started a new cadre of female health workers in 2006, the community midwives (CMWs), to improve skilled birth attendance. These females with at least ten years of education were trained for one and a half years in midwifery and deployed in their own rural communities [10]. However, maternal and newborn deaths are preventable through strengthening access to health care facilities and the provision of basic emergency obstetric and newborn care (BEmONC) services in the community by involving the CMWs [4]. CMWs work more efficiently in the rural community and offer birth care in 12% of the remote districts of Punjab, the largest province of Pakistan. An estimated 3% of births are currently attended by CMWs in rural areas [11].

Multiple issues have been previously identified that hinder the operational effectiveness of CMWs. These include job insecurity, work environment, human resources deployment issues, lack of personal training, poor infrastructure, unclear job descriptions, time constraints, lack of resources, and job satisfaction [12, 13]. Many of these issues are similar to those experienced by the rest of the healthcare system in Pakistan, and these are because of the already inefficient and fragile healthcare system facing governance and health financing challenges [14]. Identification and assessment of the challenges faced by this key cadre of community-based skilled birth attendants is crucial, as these issues need to be addressed by policy-makers. The present study aimed to evaluate the problems faced by CMWs during the provision of basic EmONC services in the province of Sindh.

## **Methods**

**Fig. 1:**
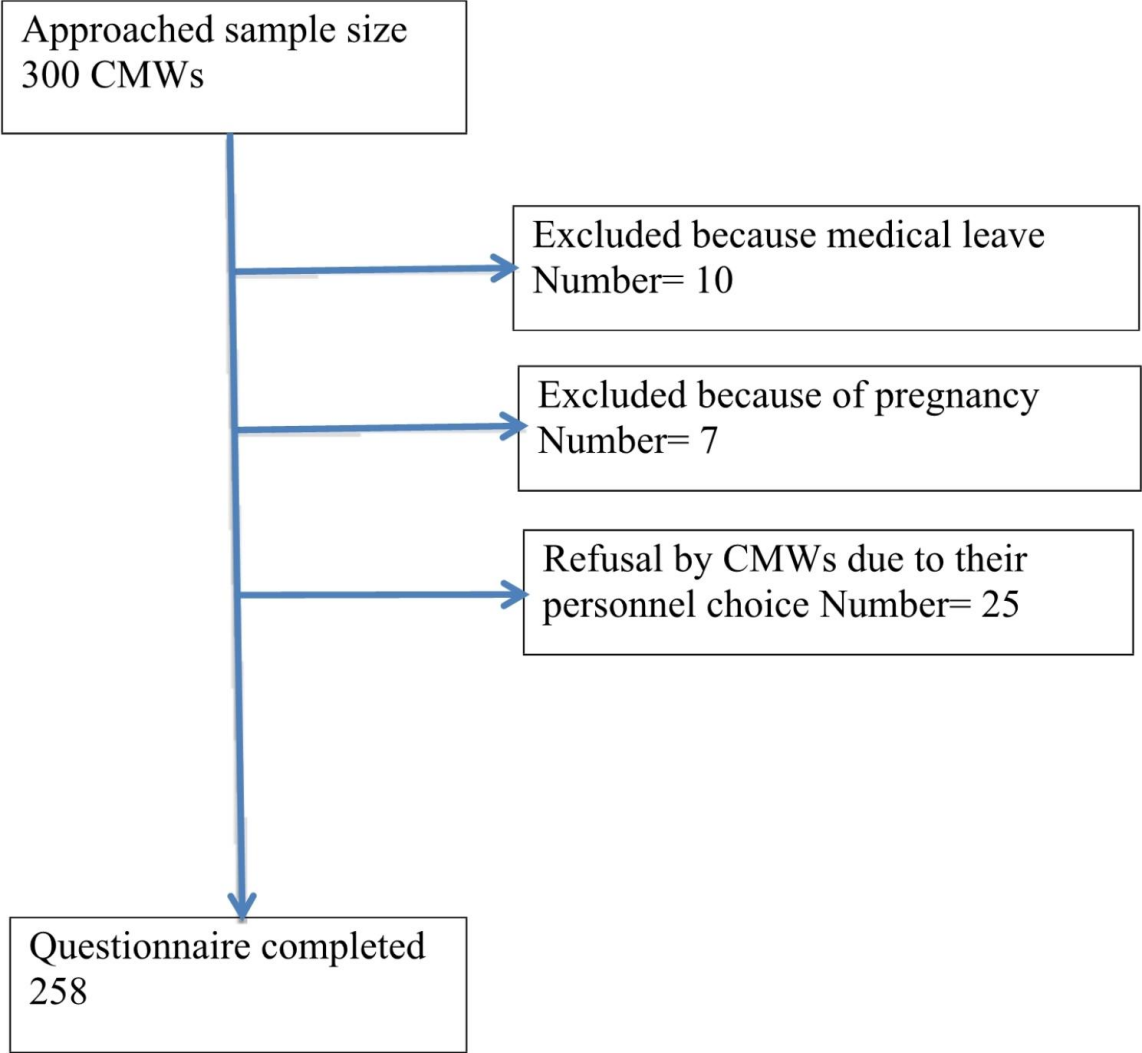
Sample Selection process

This cross-sectional study with a quantitative approach was conducted in 12 out of 29 districts in Sindh, Pakistan. These districts were selected based on poor maternal and child health indicators, according to a ranking based on the Multiple Indicator Cluster Survey [4]. A structured pretested, validated questionnaire was adopted from the previous studies [7, 11, 12]. The trained research staff, with at least graduate-level education (14 years), collected data by administering structured data collection tool to CMWs working in the selected districts. A total of 3,045 CMWs are deployed in these districts. By drawing 10% sample of the total population of CMWs working in these areas, a sample of 300 CMWs was estimated to be randomly selected from twelve districts. Next, 25 CMWs from each district were selected, using simple random sampling method, from the list of deployed CMWs available with relevant health offices and requested to participate in the study. Finally, 258 CMWs completed the questionnaire, as 42 were excluded from this study as they were on medical leave or physically too unwell to respond to the questionnaire **(**Fig. 1**)**. Besides supervising the fieldwork, the principal investigator ensured the quality of data collection by randomly validating the guided questionnaires, which were filled out at health facilities where CMWs were deployed. The data were entered in Microsoft Excel, validated, and double-checked by the supervisor and data manager.

### Variables and measurements

The outcome variable was the problems faced by CMWs while providing EmONC services in the community. These problems were categorised into three broader areas: financial, transport, and security issues. Financial difficulties were self-described by the study participants and included responses such as low salary or stipend, delay in getting a salary, no scholarship, no salary, and poor family background. Transport and accessibility difficulties were detailed by the respondents as: no pick-up and drop-off facility; no vehicle or transport for field visits; problems in the vehicle; a shortage of vehicles for field visits; scattered homes in the community; and accessibility issues in vast or far field areas. Security issues were reported as follows: law and order situation, availability of guards at hospitals, and location of health facilities.

### Data Processing and analysis

Data were entered, cleaned, and managed in Microsoft Excel and transported to statistical package of social science (SPSS) for statistical analysis. Descriptive analysis included percentage calculations for qualitative variables and mean standard deviation calculations for quantitative variables. Large numbers of categories in a variable were merged as appropriate. In inferential analysis, univariate associations between a set of covariates and the dependent variable of interest were assessed one by one at a 5% level of significance using appropriate statistical tests.

## **Results**

### General information about CMWs

Out of 258 CMWs, the majority (90%) had formal training of maternal and neonatal care from the recognized institutions. Nearly half of the respondents (43%) had working experience from one to five years, and about three-fourths (74%) had completed up to twelve years of schooling. Working hours were reported to be from six to eight hours long by half of the workers (60%), and most (85%) were available on call. Majority (81%) had direct liaison with lady health workers (LHWs) and doctors posted at a nearby health facility for patient’s referral, and more than half (62%) were in contact with the trained births attendants of the community. These CMWs were mostly deployed (60%) in upper Sindh and remaining equal proportions in lower and middle regions of the province (Table 1).

**Table 1:** Descriptive characteristics of study participant community midwives (CMW) working at the surveyed health facilities of Sindh province, Pakistan (n = 258)

Characteristic	n	%
Formal training as CMW		
Yes No	231 27	89.5 10.5
Years of working experience		
< 1 year 1–5 years > 5 years	48 112 98	18.6 43.4 38.0
Level of education		
Up to Intermediate (Grade 12) Graduate and above Not reported/Missing information	191 54 13	74.0 20.9 5.0
Duration of work at clinic/health facility		
≤ 5 h 6–10 h ≥ 11 h Not reported/Missing information	56 152 49 1	21.7 58.9 19.0 0.4
24-hour availability at work when required		
Yes No	219 39	84.9 15.1
Liaison with LHWs/Doctors in the community		
Yes No	208 50	80.6 19.4
Liaison with TBAs in the community		
Yes No	161 97	62.4 37.6
District of posting		
Lower Sindh Middle Sindh Upper Sindh	52 51 155	20.2 19.8 60.1

### Financial difficulties faced by the CMWs at their workplace

In univariate analysis, statistical differences between financial difficulties faced by CMWs and a set of correlates such as higher education were seen with having financial difficulties (p = 0.006). CMWs’ on-call availability was negatively associated with having financial difficulties (p = 0.008). Similarly, among those CMWs who were having financial difficulties, a higher proportion of them (40.5% vs. 15.7%) were posted in middle Sindh (p < 0.001). While formal training, years of working experience, longer duration of work, and liaison with health workers were found to be statistically nonsignificant in relation to financial difficulty (Table 2).

**Table 2:** Univariate association between financial difficulties faced by CMWs and set of correlates

Characteristic	Financial difficulty		
	Yes n (%)	No n (%)	P-Value
Formal training as CMW			
Yes No	37 (88.1) 5 (11.9)	194 (89.8) 22 (10.2)	0.783^$^
Years of working experience			
< 1 year 1–5 years > 5 years	12 (28.6) 15 (35.7) 15 (35.37)	36 (16.7) 97 (44.9) 83 (38.4)	0.180
Level of education			
Up to Intermediate (Grade 12) Graduate and above	26 (61.9) 16 (38.1)	165 (81.3) 38 (18.7)	0.006*
Duration of work at clinic/health facility			
≤ 5 h 6–10 h ≥ 11 h	9 (21.4) 24 (57.1) 9 (21.4)	47 (21.9) 128 (59.5) 40 (18.6)	0.912
24-hour availability at work when required			
Yes No	30 (71.4) 12 (28.6)	189 (87.5) 27 (12.5)	0.008*
Liaison with LHWs/Doctors in the community			
Yes No	37 (88.1) 5 (11.9)	171 (79.2) 45 (20.8)	0.180
Liaison with TBAs in the community			
Yes No	26 (61.9) 16 (38.1)	135 (62.5) 81 (37.5)	0.942
District of posting			
Lower Sindh Middle Sindh Upper Sinch	1 (2.4) 17 (40.5) 24 (57.1)	51 (23.6) 34 (15.7) 131 (60.6)	< 0.001*

*Significant p-value

^$^ Calculated by Fisher’s Exact Test, otherwise χ^2^test

### Transport difficulties faced by CMWs at their workplace

Further, the relationship between transport difficulties faced by CMWs and a set of correlates such as formal training (p = 0.001), working experience (p = 0.015), longer duration of work (p < 0.003), liaison with health workers, and district of posting (p = < 0.001) were found to be statistically significant. On the other hand, level of education and round-the-clock availability on call were not associated with transport difficulties faced by CMWs (Table 3).

**Table 3:** Univariate association between transport difficulties faced by CMWs and set of correlates

Characteristic	Transport difficulty		
	Yes n (%)	No n (%)	P-Value
Formal training as CMW			
Yes No	24 (68.6) 11 (31.4)	207 (92.8) 16 (7.2)	0.001*^$^
Years of working experience			
< 1 year 1–5 years > 5 years	3 (8.6) 23 (65.7) 9 (25.7)	45 (20.2) 89 (39.9) 89 (39.9)	0.015*
Level of education			
Up to Intermediate (Grade 12) Graduate and above	27 (77.1) 8 (22.9)	164 (78.1) 46 (21.9)	0.900
Duration of work at clinic/health facility			
≤ 5 h 6–10 h ≥ 11 h	2 (5.7) 20 (57.1) 13 (37.1)	54 (24.3) 132 (59.5) 36 (16.2)	0.003*
24-hour availability at work when required			
Yes No	27 (77.1) 8 (22.9)	192 (86.1) 31 (13.9)	0.169
Liaison with LHWs/Doctors in the community			
Yes No	16 (45.7) 19 (54.3)	192 (86.1) 31 (13.9)	< 0.001*
Liaison with TBAs in the community			
Yes No	12 (34.3) 23 (65.7)	149 (66.8) 74 (33.2)	< 0.001*
District of posting			
Lower Sindh Middle Sindh Upper Sinch	14 (40.0) 11 (31.4) 10 (28.6)	38 (17.0) 40 (17.9) 145 (65.0)	0.001*

*Significant p-value

^*$*^ *Calculated by Fisher’s Exact Test, otherwise χ* ^*2*^ *test*

### Security difficulties faced by CMWs at their workplace

Moreover, associations between security difficulties faced by CMWs and sets of correlates such as formal training (p = 0.019), working experience (p = 0.001), longer duration of work (p = 0.023), 24 h of availability on call (p = 0.004), liaison with TBAs in the community (p = 0.002), and district of posting (p = 0.001) were found to be statistically significant. However, education level and liaison with LHWs and doctors (= 0.118) were not associated with CMWs’ security related difficulties **(**Table 4**)**.

**Table 4:** Univariate association between security difficulties faced by CMWs and set of correlates

Characteristic	Security difficulty		
	Yes n (%)	No n (%)	P-Value
Formal training as CMW			
Yes No	39 (100.0) 0 (0.0)	192 (87.7) 27 (12.3)	0.019*^$^
Years of working experience			
< 1 year 1–5 years > 5 years	17 (43.6) 7 (17.9) 15 (38.5)	31 (14.2) 105 (47.9) 83 (37.9)	< 0.001*
Level of education			
Up to Intermediate (Grade 12) Graduate and above	31 (86.10 5 (13.9)	160 (76.6) 49 (23.4)	0.201
Duration of work at clinic/health facility			
≤ 5 h 6–10 h ≥ 11 h	13 (33.3) 24 (61.5) 2 (5.1)	43 (19.7) 128 (58.7) 47 (21.6)	0.023*
24-hour availability at work when required			
Yes No	39 (100.0) 0 (0.0)	180 (82.2) 39 (17.8)	0.004*
Liaison with LHWs/Doctors in the community			
Yes No	35 (89.7) 4 (10.3)	173 (79.0) 46 (21.0)	0.118
Liaison with TBAs in the community			
Yes No	33 (84.6) 6 (15.4)	128 (58.4) 91 (41.6)	0.002*
District of posting			
Lower Sindh Middle Sindh Upper Sinch	23 (59.0) 0 (0.0) 16 (41.0)	29 (13.2) 51 (23.3) 139 (63.5)	< 0.001*

*Significant p-value

^$^ Calculated by Fisher’s Exact Test, otherwise χ^2^test

### Other difficulties faced by CMWs at their workplace

Associations between other difficulties faced by CMWs and sets of correlates such as working experience (p = < 0.001), liaison with the traditional birth attendants in the community (p = < 0.001), and longer duration of work (p = 0.057) were found to be statistically significant. Formal training (p = 0.196), round-the-clock on call (p = 0.140), liaison with doctors or lady health workers in the community (p = 0.265), district of posting (p = 0.528) and level of education (p = 0.113) were not associated with other difficulties faced by the CMWs **(**Table 5**)**.

**Table 5:** Univariate association between other difficulties^#^ faced by CMWs and set of correlates

Characteristic	Other difficulties		
	**Yes** **n (%)**	**No** **n (%)**	**P-Value** ^$^
Formal training as CMW			
Yes No	74 (86.0) 12 (14.0)	157 (91.3) 15 (8.7)	0.196
Years of working experience			
< 1 year 1–5 years > 5 years	6 (7.0) 54 (62.8) 26 (30.2)	42 (24.4) 58 (33.7) 72 (41.9)	< 0.001*
Level of education			
Up to Intermediate (Grade 12) Graduate and above	64 (84.2) 12 (15.8)	127 (75.1) 42 (24.9)	0.113
Duration of work at clinic/health facility			
≤ 5 h 6–10 h ≥ 11 h	25 (29.4) 42 (49.4) 18 (21.2)	31 (18.0) 110 (64.0) 31 (18.0)	0.057
24-hour availability at work when required			
Yes No	69 (80.2) 17 (19.8)	150 (87.2) 22 (12.8	0.140
Liaison with LHWs/Doctors in the community			
Yes No	66 (76.7) 20 (23.3)	142 (82.6) 30 (17.4)	0.265
Liaison with TBAs in the community			
Yes No	40 (46.5) 46 (53.5)	121 (70.3) 51 (29.7)	< 0.001*
District of posting			
Lower Sindh Middle Sindh Upper Sinch	15 (17.4) 20 (23.3) 51 (59.3)	37 (21.5) 31 (18.0) 104 (60.5)	0.528

^*#*^ *Bad behavior of senior management or community members, language problems, lack of awareness about health in the community, family issues, sexual harassment, lack of training, overwork, missing equipment for delivery, medicine shortage, complicated cases, rod problem, administrative issues such as frequent changes in duty timings, transfer due to political influence and government job confirmation*

*Significant p-value

^*$*^ *Calculated by χ* ^*2*^ *test*

Findings show that financial, transportation, and security issues are major problems faced by the CMWs while providing basic EmONC services, and factors like formal training, longer duration of work, liaison with health workers, and posting district were found to be statistically significant about transportation issues. Security difficulties faced by CMWs and a set of correlates such as formal training, working experience, longer duration of work, 24 h of availability on call, liaison with TBAs in the community, and district of posting were found to be statistically significant. Other issues, like working experience and liaison with TBAs in the community, were found to be statistically significant.

## Discussion

This research highlights the issues faced by CMWs during the provision of basic EmONC at the community level to establish a successful practice of midwifery in rural Pakistan and improve skilled birth care. The major constraints identified were financial, transport, and security related hindrances; and these were found statistically significant. However, CMWs continue their work despite facing these challenges to sustain their services in the community. CMWs may only have control over their professional work, job-related skills, and provision of maternal care and childbirth with dignity and honor.

In this study, financial constraints were found to be positive correlates with level of education, location of posting, and availability at job. Our study also support our findings that financial incentives for health workers could enhance the uptake and delivery of maternal care services [15]. A previous study supports the current finding that qualified workers are less likely to be willing to work in rural areas due to poor incentives [13]. Supportive findings suggested those employees are available at work round-the-clock and require financial incentives in the form of overtime payments or special pay packages to ensure their motivation to continue to work in rural settings [14].

CMWs in our study faced transport related challenges that significantly impact upon their professional training, work experience, round-the-clock availability and coordination with other health workers in the community. Studies supports our finding and show that the training of healthcare workers is mandatory for refreshing the knowledge and skills of employees and need to be offered regularly [11]. Moreover, study also supports that the EmONC refresher training also appears to improve the skills of healthcare workers and achieve better health outcomes [16]. Availability of healthcare workers and their access to health facilities is linked with better quality of maternal and birth healthcare services [13, 14]. A lack of geographical and access by vehicles are major hindrances to accessing facility-based maternal care [17]. Our findings are consistent with other studies that show that the health providers who have better access to their workplace or those who are incentivised for their transport are more likely to be motivated. It is also evident that the behaviour of both patients and providers is positively influenced by the provision of incentives in the health system [11, 14, 18].

Security issues faced by CMW were also found to be statistically significant with training, work experience, job duration, and coordination with health workers in this study. The literature also identifies several barriers to providing services by CMWs that are similar to the findings from our study [19]. Previous research also supports our findings that poor resources and insecurity are major issues in the provision of maternal healthcare in already fragile health systems in rural and remote areas [20, 21]. Research from other settings supports our findings that job security, work environment, and time constraints positively motivate healthcare workers in any organization [13]. Previous research also supports our findings that CMWs face security related issues during their work in the community [22]. A study showed that health workers are reluctant to serve in insecure areas; and because of this, inadvertently, these communities commonly practice home births resulting in high risks to maternal and fetal health and survival [23]. Our findings are consistent with a study that found female healthcare workers and patients were hesitant to visit health facilities alone and needed to be accompanied by a member of their family to avoid any untoward security issues (24). It is therefore important that the CMWs are deployed near their homes. This is supported by the evidence showing that health providers feel more secure if posted near their place of living as compared to those who are posted far [17]. Moreover, findings show the safe security at health facilities established in rural areas can prevent workers to serve there [20].

## **Conclusions**

In conclusion, financial, transportation, and security issues are major hindrances for CMWs in providing basic EmONC services in the rural areas of the country, where the burden of maternal and child health mortality and morbidity is higher than in urban areas. It is recommended that these problems need to be considered and addressed by policymakers to improve maternal and child health in Pakistan.

## Data Availability

The datasets used and/or analyzed during the current study are available from the corresponding author upon reasonable request. The data are not publicly available due to privacy and ethical restrictions.

## Abbreviations

CMWs: Community midwives
EmONC: Emergency obstetric and neonatal care
WHO: World Health Organization
CMWs: Community midwives
BEmONC: Basic Emergency obstetric and neonatal care
LHWs: Lady Health workers
TBAs: Traditional Birth attendants
WHO: World Health Organization
